# Investigating frailty and quality of life in patients with heart failure and CKD (FRAIL study)

**DOI:** 10.1002/ehf2.15096

**Published:** 2024-09-19

**Authors:** Izabella Uchmanowicz

**Affiliations:** ^1^ Department of Nursing, Faculty of Nursing and Midwifery Wroclaw Medical University Wroclaw Poland; ^2^ Centre for Cardiovascular Health Edinburgh Napier University Edinburgh UK

## Introduction

The intersection of chronic kidney disease (CKD) and heart failure (HF) represents a significant clinical challenge due to the complex bidirectional relationship between these conditions.^1^ As the prevalence of multimorbidity rises, particularly in ageing populations, understanding the interactions between various chronic conditions and their impact on patients' lives is increasingly important. Frail patients with HF and CKD experience worse treatment outcomes compared to their non‐frail counterparts, particularly regarding hospitalization and mortality rates. Frailty can significantly diminish patients' health‐related quality of life (HRQOL), primarily due to factors such as increased fatigue and impaired cognitive function. HRQOL is a crucial outcome measure in patients with both CKD and HF, given the complexity of their management and the challenges in assessing the impact of treatment decisions on their lives.

Quality of life (QoL) is increasingly recognized as a vital aspect of patient care, particularly for those with chronic and debilitating conditions such as CKD and HF.^2^, ^3^ QoL encompasses physical, psychological and social domains of health, which are significantly impacted by these chronic illnesses. For patients, maintaining a satisfactory QoL often holds as much importance as the clinical outcomes of their treatments. Addressing QoL can lead to better overall patient satisfaction, adherence to treatment regimens and improved clinical outcomes.^4^, ^5^

The FRAIL study, conducted by McNally et al., significantly contributes to this understanding by investigating the prevalence of frailty and its impact on QoL in patients with concurrent CKD and HF.^6^ The study's primary aim was to elucidate how frailty, a known predictor of morbidity and mortality, affects the QoL of patients suffering from both CKD and HF. Using robust tools such as the Modified Frailty Phenotype (MFP) and the Medical Outcomes Study 36‐item Short Form Health Survey (SF‐36), the researchers provided valuable insights into the lives of these patients.^6^ One of the most salient findings of this study is the elevated prevalence of frailty within the cohort, with nearly half of the participants classified as frail. This prevalence is markedly higher than that observed in the general population, underscoring the compounded vulnerability of patients with coexisting CKD and HF. Similar trends have been identified in other patient groups suffering from heart failure and CKD independently, making these findings anticipated when the two comorbidities converge.^7^, ^8^, ^9^

Recent studies further highlight the critical impact of frailty on patient outcomes. For instance, Yamamoto et al. found that cognitive frailty significantly affects prognosis in older adults with heart failure, emphasizing the need for comprehensive assessments.^10^ Additionally, Guasti et al. demonstrated the potential of digital health interventions in managing frailty among older adults, providing a promising avenue for improving QoL.^11^ Roehrich et al. compared various frailty interventions, underscoring the importance of tailored approaches to enhance feasibility and outcomes in frail populations.^12^

The study by McNally et al. found that frail patients reported significantly poorer QoL across several domains, including physical functioning, general health, bodily pain, social functioning and energy levels.^6^ This multifaceted impact on QoL highlights the need for healthcare providers to prioritize the early identification and management of frailty in CKD‐HF patients. It is crucial to remember that assessing frailty is important at every stage of the HF trajectory. Frailty should be evaluated in patients with newly diagnosed HF, and a standard assessment should be conducted during periods of clinical stabilization. Given the fluctuating clinical conditions of HF patients, periodic re‐assessment of frailty features is recommended.^13^, ^14^

The FRAIL study also sheds light on the barriers to optimal care for CKD‐HF patients.^6^ Frailty was found to be associated with polypharmacy, reflecting the complexities of managing multiple chronic conditions simultaneously. While polypharmacy can be necessary, it also increases the risk of adverse drug interactions, reduced medication adherence and additional strain on patients' physical and cognitive resources. It is important to monitor patients, particularly the elderly with frailty and multimorbidity, for potential adverse effects related to pharmacotherapy, as these complications may be more common in this vulnerable population.^15^ The study reveals a significant misalignment between healthcare providers' priorities and those of the patients. While patients prioritized better QoL over longer survival or reduced hospital admissions, healthcare providers often focus on clinical outcomes such as prolonging life and minimizing hospital stays. This discrepancy calls for a paradigm shift towards more patient‐centred care, where improving QoL is integrated into the primary goals of treatment plans.^6^ The findings from the FRAIL study underscore the importance of incorporating frailty assessments into routine clinical practice for CKD‐HF patients. Frailty negatively impacts the health‐related quality of life (HRQOL) in individuals with HF.^16^

Hamada et al.'s findings suggest that physical frailty significantly affects the implementation of guideline‐directed medical therapy (GDMT) in heart failure patients with reduced ejection fraction. This is particularly relevant to the FRAIL^17^ study as it underscores the complexity of managing frail patients, who are less likely to receive full therapeutic regimens due to their frailty. The lower implementation rates of GDMT among frail individuals could explain part of the observed poorer outcomes and quality of life in these patients.

Early detection of frailty can facilitate timely interventions that may improve both clinical outcomes and QoL. Interventions could include tailored exercise programmes, nutritional support and comprehensive management plans that address both the physical and psychological aspects of frailty.

Further research is needed to develop and validate interventions specifically designed for frail patients with CKD‐HF. Longitudinal studies that track the impact of such interventions on both frailty and QoL over time will be crucial in establishing evidence‐based practices.

## Conclusions

The FRAIL study by McNally et al. is a pivotal piece of research that provides essential insights into the relationship between frailty and QoL in patients with CKD and HF. It emphasizes the critical need for healthcare systems to adopt a more holistic, patient‐centred approach that prioritizes the overall well‐being of patients, alongside traditional clinical outcomes. As the population ages and the burden of multimorbidity grows, such insights will be invaluable in shaping the future of chronic disease management.

## References

[1] Xanthopoulos A , Papamichail A , Briasoulis A , Loritis K , Bourazana A , Magouliotis DE , *et al*. Heart failure in patients with chronic kidney disease. J Clin Med 2023;12:6105. doi:10.3390/jcm12186105 37763045 PMC10532148

[2] Sharma S , Kalra D , Rashid I , Mehta S , Maity MK , Wazir K , *et al*. Assessment of health‐related quality of life in chronic kidney disease patients: a hospital‐based cross‐sectional study. Med Kaunas Lith 2023;59:1788. doi:10.3390/medicina59101788 PMC10608694 37893506

[3] Lawson CA , Benson L , Squire I , Zaccardi F , Ali M , Hand S , *et al*. Changing health related quality of life and outcomes in heart failure by age, sex and subtype. eClinicalMedicine 2023;64:102217. doi:10.1016/j.eclinm.2023.102217 37745020 PMC10514432

[4] Kefale B , Alebachew M , Tadesse Y , Engidawork E . Quality of life and its predictors among patients with chronic kidney disease: a hospital‐based cross sectional study. PLoS ONE 2019;14:e0212184. doi:10.1371/journal.pone.0212184 30811447 PMC6392259

[5] Haraldstad K , Wahl A , Andenæs R , Andersen JR , Andersen MH , Beisland E , *et al*. A systematic review of quality of life research in medicine and health sciences. Qual Life Res 2019;28:2641‐2650. doi:10.1007/s11136-019-02214-9 31187410 PMC6761255

[6] McNally T , Tumelty E , Chung I , Hussain S , Mookerjee S , Ali MA , *et al*. Investigating the relationship between FRailty And Quality of LIfe in patients with heart faiLure and CKD (FRAIL study). ESC Heart Fail 2024;11:1411‐1421. doi:10.1002/ehf2.14693 38320815 PMC11098643

[7] Vitale C , Jankowska E , Hill L , Piepoli M , Doehner W , Anker SD , *et al*. Heart Failure Association/European Society of Cardiology position paper on frailty in patients with heart failure. Eur J Heart Fail 2019;21:1299‐1305. doi:10.1002/ejhf.1611 31646718

[8] Vitale C , Uchmanowicz I . Frailty in patients with heart failure. Eur Heart J Suppl J Eur Soc Cardiol 2019;21:L12‐L16. doi:10.1093/eurheartj/suz238 PMC6926418 31885506

[9] Denfeld QE , Winters‐Stone K , Mudd JO , Gelow JM , Kurdi S , Lee CS . The prevalence of frailty in heart failure: a systematic review and meta‐analysis. Int J Cardiol 2017;236:283‐289. doi:10.1016/j.ijcard.2017.01.153 28215466 PMC5392144

[10] Yamamoto S , Yamasaki S , Higuchi S , Kamiya K , Saito H , Saito K , *et al*. Prevalence and prognostic impact of cognitive frailty in elderly patients with heart failure: sub‐analysis of FRAGILE‐HF. ESC Heart Fail 2022;9:1574‐1583. doi:10.1002/ehf2.13844 35182038 PMC9065815

[11] Guasti L , Dilaveris P , Mamas MA , Richter D , Christodorescu R , Lumens J , *et al*. Digital health in older adults for the prevention and management of cardiovascular diseases and frailty. A clinical consensus statement from the ESC Council for Cardiology Practice/Taskforce on Geriatric Cardiology, the ESC Digital Health Committee and the ESC Working Group on e‐Cardiology. ESC Heart Fail 2022;9:2808‐2822. doi:10.1002/ehf2.14022 35818770 PMC9715874

[12] Roehrich L , Sündermann SH , Just IA , Kopp Fernandes L , Stein J , Solowjowa N , *et al*. Comparison of feasibility and results of frailty assessment methods prior to left ventricular assist device implantation. ESC Heart Fail 2022;9:1038‐1049. doi:10.1002/ehf2.13764 34994094 PMC8934953

[13] Martín‐Sánchez FJ , Christ M , Miró Ò , Peacock WF , McMurray JJ , Bueno H , *et al*. Practical approach on frail older patients attended for acute heart failure. Int J Cardiol 2016;222:62‐71. doi:10.1016/j.ijcard.2016.07.151 27458825 PMC5731778

[14] Wleklik M , Denfeld Q , Czapla M , Jankowska EA , Piepoli MF , Uchmanowicz I . A patient with heart failure, who is frail: how does this affect therapeutic decisions? Cardiol J 2023;30:825‐831. doi:10.5603/CJ.a2023.0027 37067336 PMC10635718

[15] Jankowska EA , Vitale C , Uchmanowicz I , Tkaczyszyn M , Drozd M , Ponikowski P . Drug therapy in elderly heart failure patients. Eur Heart J Suppl J Eur Soc Cardiol 2019;21:L8‐L11. doi:10.1093/eurheartj/suz237 PMC6926409 31885505

[16] Buck HG , Riegel B . The impact of frailty on health related quality of life in heart failure. Eur J Cardiovasc Nurs 2011;10:159‐166. doi:10.1016/j.ejcnurse.2010.06.001 20587372

[17] Hamada T , Kubo T , Kawai K , Nakaoka Y , Yabe T , Furuno T , *et al*. Frailty interferes with the guideline‐directed medical therapy in heart failure patients with reduced ejection fraction. ESC Heart Fail 2023;10:223‐233. doi:10.1002/ehf2.14163 36193578 PMC9871689

